# Preparation of Mesoporous Biochar from Cornstalk for the Chromium (VI) Elimination by Using One-Step Hydrothermal Carbonation

**DOI:** 10.1155/2021/3418887

**Published:** 2021-10-05

**Authors:** Chao Wang, Jun Xie, Mingdong Zheng, Jinbo Zhu, Changliang Shi

**Affiliations:** ^1^Department of Materials Science and Engineering, Anhui University of Science & Technology, Huainan, China; ^2^College of Chemistry and Chemical Engineering, Henan Polytechnic University, Jiaozuo, China

## Abstract

Hydrothermal carbon (HTC) was prepared by the one-step hydrothermal method for Cr (VI) removal from wastewater, which was considered a “green chemistry” method. The specific surface area (S_BET_) of HTC was 85 m^2^/g with the pore size in range of 2.0–24.0 nm. FT-IR spectra analysis showed that the HTC had abundant chemical surface functional groups. The influence of adsorption parameters such as pH, HTC dosage, Cr (VI) concentration, and contact time on the removal efficiency of Cr (VI) had been investigated. When the initial concentration was 50 mg/L, pH = 6, amount of adsorbent was 0.2 g/50 ml, and adsorption time was 90 min; the Cr (VI) absorbed rate of HTC reached 98%. Batch adsorption experiments indicated that Cr (VI) adsorption data of HTC fitted the Freundlich isothermal and pseudo-second-order kinetic models. Overall, our findings provide a promising material in treatment of Cr (VI)-rich wastewater and give a clear picture of its application, which is worthy of further study.

## 1. Introduction

Heavy metal contamination in water is becoming a concerning global environmental issue. Heavy metal pollutants, such as chromium, arsenic, cadmium, nickel, copper, and lead, are difficult to be removed or degraded from the water mostly due to their high stability [1]. Among these heavy metals, chromium was noticed as a hazardous pollutant introduced by multiple industrial processes including electroplating, leather tanning, pigment, and production paint [2]. In wastewater, chromium existed principally with two comparatively stable forms including hexavalent Cr (VI) and trivalent Cr (III) [3, 4], in which Cr (VI) was relatively hazardous than Cr (III), especially in the human body [5, 6]. Moreover, Cr (VI) revealed a nature of absorption and accumulation within human bodies such as the stomach, kidneys, and liver especially, which caused the severe somatic damages [5, 7–9].

Massive efforts focused on precipitation, electrochemical recovery, solvent extraction, membrane separation, and ion exchange had been devoted to eliminating Cr (VI) from industry wastewaters [10–14]. However, numerous approaches were indicated as invalid and expensive, which led to secondary pollution to the environment as a consequence in some cases [15]. Adsorption had been regarded as the most effective method aiming to eliminate the contaminants within the aqueous systems [16]. Hence, the investigation in appropriate adsorbent emerged the importance of developing a considerable adsorption technology [16, 17]. Activated carbons have better adsorption capacity and Cr (VI) absorbed rate resulted from the large surface area and volume, which have been widely employed to eliminate Cr (VI) ions from wastewater resulted from its low cost, high adsorption efficiency, and simple operation [18, 19]. Unfortunately, activated carbons are commonly prepared via chemical or physical activation methods. In general, biomass materials were employed as precursors [20], which require high energy consumption and cause damage to the environment [21–23]. Therefore, it is particularly important to find a synthesis method with low energy consumption. Recently, hydrothermal carbonization (HTC) processes as a nascent technology to produce functional materials was reported resulting from its low cost, simplicity in operation, and high energy efficiency [24, 25]. One was also be classified as “green” because no organic solvents or surfactants were needed in the treatment process [25].

Biochar is produced from agriculture and forest waste which contain mainly carbon. Biochar-type materials have raised increasing attention attributed to their unique mesoporous structure, high ion exchange capacity, and high specific surface which allow them to be widely used for applications in greenhouse gas reduction, soil improvement, and remediation of contaminated soil [26–28]. Guo et al. reported the Cr (VI) adsorption on the HTC prepared from rice husk at 650–850°C and found adsorption efficiency is related to the micoporous and mesoporous [29]. Anandkumar and Mandal used AC prepared from bael fruit at 600°C to eliminate Cr (VI) with a considerable value of 17.27 mg/g in adsorption capacity that had been acquired [30]. Also, several activated carbon adsorbents from agricultural and biological wastes including almond shells, straw, waste tea, coconut shells, cactus leaves [31, 32], and algae had been introduced in chromium (VI) elimination from water solutions. However, the production process of biochar is relatively complex. The biomass was first preheated at low temperature and followed by chemical activation. Last, the biomass was hydrothermally carbonized at high temperatures such as 600, 800, and even 1000°C.

In this work, the mesoporous biochar was acquired using one-step hydrothermal carbonization from cornstalk at relatively mild temperature conditions (190°C) at self-generated pressures. The effects of several important operating parameters on the removal of Cr (VI) from aqueous solution, such as pH, adsorption dosage, initial concentration of the solution, and contact time, were studied by batch experiments. Furthermore, the adsorption kinetics and isotherms of Cr (VI) on HTC at different temperatures were also analyzed and discussed.

## 2. Experimental

### 2.1. Materials

**Figure 1:**
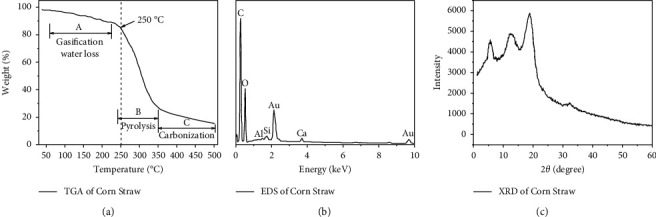
The TGA (a), EDS (b), and XRD (c) curves of corn straw.

The raw material of this experiment is corn straw, which was collected in Huainan City, Anhui Province. TGA, EDS, and XRD of corn straw are shown in Figures 1(a), 1(b), and 1(c), respectively. According to the TGA curve, the weight loss of corn straw can be divided into three stages: A (gasification stage), B (thermal cracking stage), and C (carbonization stage). The decomposition temperature exceeds 200°C and has thermal stability. The characteristic peaks of C and O elements in EDS showed that the main component of corn straw was cellulose. The XRD diffraction pattern shows that the diffraction peak is 14.8°, 16.5°, and 22.5°, representing (101) and (002) crystal planes of cellulose type I crystal structure, respectively, indicating that the crystal structure has not changed. Cornstalk was acquired from local natural resources and was cleaned and dried to constant weight at room temperature. Sulfuric acid (98 wt.%), phosphoric acid, sodium hydroxide, acetone, potassium dichromate, and analytical grade 1,5-diphenylcarbazide were acquired from Beijing Chemical Reagents Company. Potassium dichromate (K_2_Cr_2_O_7_) with a certain quantity was dissolved into deionized water to prepare the stock solution and then diluted to the required concentration for further analysis.

### 2.2. Synthesis of HTC

**Figure 2:**
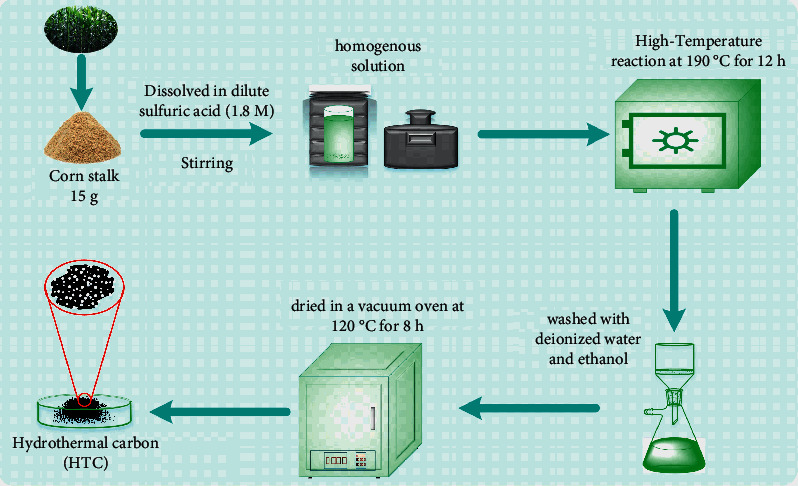
Synthesis process of the HTC.

Different synthesis methods of HTC were considered [32–34]. The preparation of biomass porous carbon usually requires a high temperature calcination process [21–23], while our hydrothermal carbon (HTC) is prepared by a one-step hydrothermal method, which is much simpler. A representative process is selected, as shown in Figure 2. 15 g corn stalk had been dissolved into 80 mL of dilute sulfuric acid (1.84 M) with stirring to form a homogenous solution. The mixture was subsequently removed to 120 mL Teflon-lined stainless steel autoclave and then placed in an electronic heating furnace (preheated at 190°C) for 12 h. After cooled to room temperature, the solid product was gathered by a filter flask and cleaned by a deionized ethanol solution. HTC products had been dried in a vacuum oven at 120°C for 8 h.

### 2.3. Characterization Techniques

The porous texture of the HTC was analyzed by N_2_ sorption at −196°C, using an automatic adsorption system (Quanta Chrome, America). Before measurements, the sample was degassed at 120°C for 5 h. The specific surface area of the sample was calculated by the Brunauer–Emmett–Teller (BET) method using the adsorption data at the relative pressure (*P*/*P*_*o*_) range of 0.05–0.3. The total pore volume was calculated at *P*/*P*_o_ = 0.99, and the pore size distribution curve was computed using the BJH model.

Infrared spectra (5000–0 cm^−1^) were recorded using a Bruker VERTEX 70 FT-IR spectrometer. The sample was prepared by mixing an oven-dried (at 105°C) sample with spectroscopy-grade KBr in an agate mortar.

The HTC sample was dried in a fan-forced oven under air at 80°C. About 0.1-0.2 g of each sample was weighed into tin foil cups and combusted with an oxygen catalyst at 1150°C. The ultimate analyses were conducted on a Thermo Scientific FLASH 2000 autoanalyzer.

Powdered HTC sample was placed onto the adhesive carbon tape on an aluminum stub followed by sputter coating with gold. The surface morphology of the sample was observed on a UL-TRA55 scanning electron microscope (SEM) operated at 2 kV.

### 2.4. Adsorption Experiments

Adsorption characteristic had been achieved under the batch mode at 35°C. First, a certain amount of 1 M HCl and 1 M NaOH had been applied to modify the pH value of each solution followed by mixed with the adsorbent. The influence of pH on the adsorbability of HTC had been illustrated to determine an optimal pH value. The effects of initial adsorbate concentration, dosage, and contacting duration on the adsorption performance were then studied at the optimum pH. The adsorbent was employed to mix with 50 mL of adsorbate solutions with a particular initial concentration. Specimens were designed to be collected at different intervals of time, and the adsorbents were extracted by filtration. Whereafter, the filtrates had been investigated in the residuary chromium (VI) concentration with the UV spectrophotometer (TU-1880, Beijing, China) under the wavelength of 540 nm. The amount of adsorbed had been evaluated via the following equation:

(1) $$\begin{array}{c} q=\frac{v\left(c_{0}-c_{t}\right)}{m}, \end{array}$$

where *q* is the amount of Cr (VI) ions adsorbed per unit gram of hydrothermal carbon (mg/g) at any time (*t*), *c*_*t*_ is the final Cr (VI) concentration after a certain period (mg/L), *c*_0_ is the initial Cr (VI) concentration (mg/L), and *v* is the initial solution volume (L); *m* is the HTC mass (g). The percentage of removed metal ions in the solution was calculated using the following equation:

(2) $$\begin{array}{c} \eta =\frac{\left(c_{0}-c_{t}\right)}{c_{0}}. \end{array}$$

## 3. Results and Discussion

### 3.1. Pore Structure Characterization

**Figure 3:**
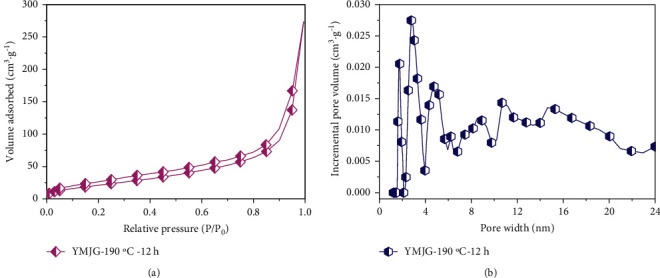
N_2_ adsorption-desorption isotherms (a) and pore sizes distribution curves (b) of YMJG.

The curves of the pore size distribution (PSD) and corresponding nitrogen adsorption-desorption isotherm for HTC samples are shown in Figure 3. According to the IUPAC classification, it can be seen from Figure 1(a) that the HTC exhibits a type IV isotherm curve. There was no significant increase in nitrogen uptake when the relative pressure was below 0.01, the apparent increase in nitrogen adsorption at the range from 0.1 to 1.0 in relative pressure, and the capillary hysteresis loop indicates the existence of developed mesoporous structure in the sample.  Figure 3(b) shows its pore size distribution in 2.0–24.0 nm, in which multiple peaks are shown, indicating that the HTC has a broad size distribution.  Table 1 provides the textural characteristics of the HTC which exists an S_BET_ and overall pore volume (*V*_t_) of 85 m^2^/g, 0.042 cm^3^/g, respectively. These values are even higher than the reports in some kinds of literature [35, 36] at higher hydrothermal temperatures. The large S_BET_ and broad distribution of pore size is necessary for the Cr (VI) ions reaching the interior of the material, thus achieving maximum absorption.

### 3.2. FT-IR Spectra and Ultimate Analyses of the HTC-Cornstalk

**Figure 4:**
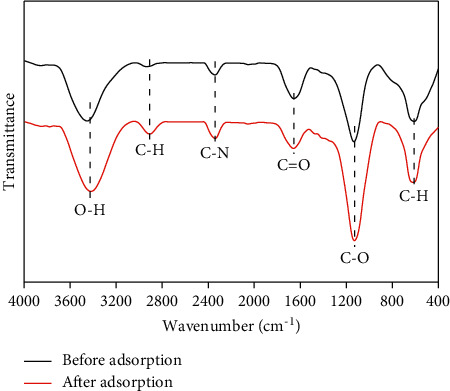
FT-IR spectra of the HTC-cornstalk.

Structure analysis of the HTC-cornstalk at the initial and end states of the adsorption had been revealed via FT-IR spectra and is shown in Figure 4. O-H (bonded) stretching vibration is correlative to the band between 3700 and 3030 cm^−1^. Moreover, peaks of 2929 and 2376 cm^−1^ implied the stretching vibration of C-H and C-N, respectively, while 1700 and 1600 cm^−1^ were associated with C = O and C = C stretching [36]. The absorption band between 995 and 1242 cm^−1^ is attributed to the C-O stretching vibration of esters, aliphatic, or alcohols [37]. Band at 620 cm^−1^ was corresponded to the aromatic ring (C-H) bending vibration [38]. After adsorption, the peaks at 3450, 2929, and 2376 cm^−1^ shift towards a lower wavenumber of 3429, 2918, and 2355 cm^−1^, respectively. The absolute values of bands between 1700 and 1600 cm^−1^ are smaller after adsorption. This suggests that there were possible interactions between these groups and Cr (VI). However, the absolute values of the band at 1112 and 640 cm^−1^ become larger, which indicates that the HTC-cornstalk has higher functional groups after Cr (VI) adsorption. One is given in Table 2, in which the carbon content was increased during the HTC process, whereas the oxygen and hydrogen contents were decreased, which is consistent with other reports [39, 40].

### 3.3. SEM Analysis

**Figure 5:**
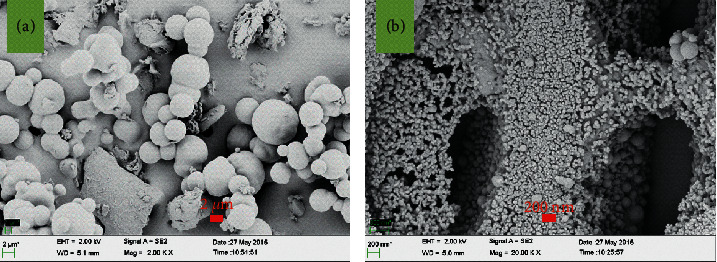
SEM images of HTC-cornstalk.

The SEM images of HTC-cornstalk at different magnifications are shown in Figures 5(a) and 5(b), respectively. It can be observed that the uniform spheres were formed on the surface of the sample. This may be because the HTC synthesized from cornstalk was not completely carbonized [41]. This phenomenon is interesting due to fact that some porosity structures could be formed during the hydrothermal carbonization of biomass materials under common conditions [41–43].  Figure 5(a) shows that there are some pores formed on the surface of the sample. Besides, many small spheres cover the inner wall of the aperture (Figure 5(b)). The reason is that the cornstalk underwent isomerization, fragmentation, dehydration, polymerization, and carbonization to form the spheres on the surface of the sample [44].

### 3.4. Batch Adsorption Studies

#### 3.4.1. Effect of pH and HTC-Cornstalk Dosage

**Figure 6:**
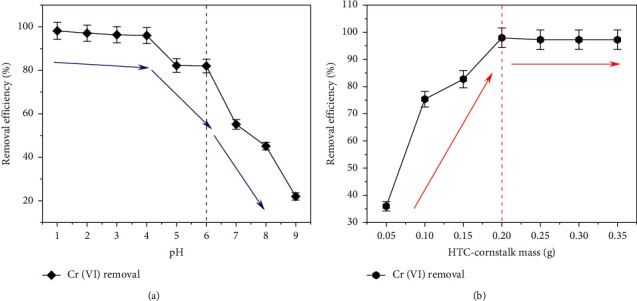
Effect of initial solution pH on the removal efficiency of Cr (VI) from aqueous solutions. (a) Volume, 50 mL; agitation speed, 120 rpm; HTC dosage, 4 g/L; effect of initial solution of the HTC dosage on the removal efficiency of Cr (VI) from aqueous solutions. (b) pH, 6; volume, 50 mL; agitation speed, 120 rpm; Cr (VI) concentration, 50 mg/L; contact time, 90 min.

At the range of 1.0–9.0 in pH values of the solution, Cr (VI) absorption induced by HTC at 35°C had been observed.  Figure 6(a) uncovers the impact of the pH value on Cr (VI) adsorption under the conditions of 50 mg/L in the initial Cr (VI) concentration and 90 min in the contacting duration. The adsorbability sharply decreases with the increase of the pH value from 4.0 to 9.0 which implied the strong correlation between adsorbing behaviors of Cr (VI) and the pH value of the solution. Only 22% Cr (VI) ions had been eliminated in the case of pH = 9 instead of over 82% while in a slightly acidic solution with pH = 6.0. Moreover, an even higher removal rate of the Cr (VI) ions of 98% had been achieved when the pH value was 1.0. The surface functional groups in the adsorbent and metal solution chemistry are highly related to the pH, which can largely affect metal adsorption ability [40, 45, 46]. Under the strong acid condition, the removal rate of Cr (VI) ion was higher; however, it would bring acid pollution to the environment. Thus, the optimum pH for the adsorption experiment is chosen as 6.

The effect of the adsorbent dosage was also investigated at 35°C, as shown in Figure 6(b). Results indicated that the removed Cr (VI) ions quantity was enlarged as the increasing adsorbent dosage. Increasing the amount of HTC-cornstalk from 0.05/50 to 0.2/50 (g/mL) largely enhanced the elimination percentage of Cr (VI) ions from 36% to 98%, resulted from the increase of the active sites which were available for the occupation of Cr (VI) ions. A further increase in the adsorbent dosage did not have any effect.

#### 3.4.2. Effect of Initial Cr (VI) Concentration and Contact Time

**Figure 7:**
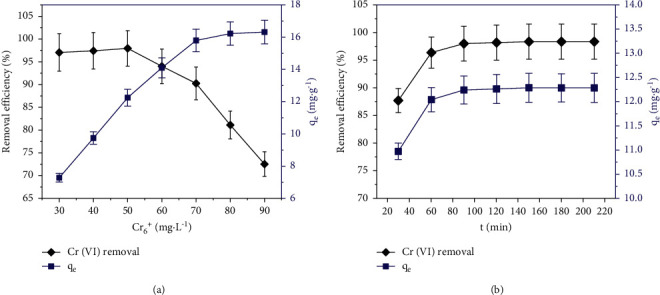
Effect of initial Cr (VI) concentration (a) and contacting duration (b) on the Cr (VI) adsorbability of HTC.

The adsorption performance of the HTC-cornstalk influenced by the initial Cr (VI) concentrations from 30 to 90 mg/L was studied at 35°C with pH = 6. Results are shown in Figure 7(a). The HTC-cornstalk dosage and the contacting duration were kept at 0.2 g/50 mL, 90 min, respectively. One was introduced that the removal efficiency and adsorbability of HTC increased along with the increase of Cr (VI) concentration. However, while the Cr (VI) concentration hit over 50 mg/L, the removal efficiency steadily reduced as a consequence. With a high Cr (VI) ions concentration, the adsorption rate was restricted because the adsorption was saturated and the desorption rate was higher than adsorption [47].

Figure 7(b) shows the impact of contacting duration on the Cr (VI) adsorption within the aqueous solution with 50 mg/L in the initial Cr (VI) concentration under the same conditions. One was cleared that the adsorption procedure approached the equilibrium state within 150 min or less. The minor effect would appear even by further increasing the contacting duration. Desorption ratio of Cr (VI) ions and adsorption quantity in 150 min was reached almost 98%, 12.29 mg/g, respectively.

### 3.5. Adsorption Kinetic Analysis

**Figure 8:**
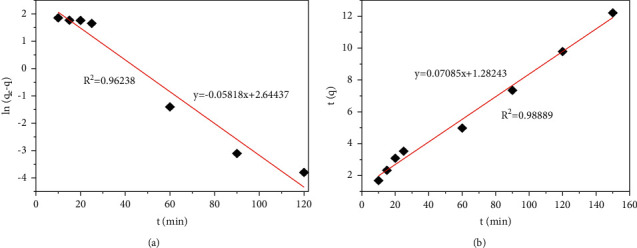
Pseudo-first-order kinetics (a) and pseudo-second-order kinetics (b) for Cr (VI) adsorption onto HTC at 35°C (initial Cr (VI) concentration 50 mg/L).

A kinetic study on adsorption was carried out to reveal the adsorption rates and the controlling adsorption mechanism. Pseudo-first-order (equation (3)) and pseudo-second-order modes (equation (4)) are commonly used to fit experimental data [48]:

(3) $$\begin{array}{c} \ln\left(q_{e}-q\right)=\ln\;q_{e}-k_{1}t, \end{array}$$

(4) $$\begin{array}{c} \frac{t}{q}=\frac{1}{k_{2}q_{e}q_{e}}+\frac{1}{q_{e}}, \end{array}$$

where *k*_1_ is the rate constant of the pseudo-first-order model (min^−1^), *k*_2_ is the rate constant of the pseudo-second-order model (g/mg. min), and *q*_*e*_ and *q* are the values of the amount adsorbed per unit mass at equilibrium and at any time *t*, respectively. The experimental data and the fitting by the two equations are shown in Figure 8, and the fitted kinetic parameters are given in Table 3.

One emerged in Figure 8 and Table 3 that both models were well-fitted with the experimental data. Furthermore, the calculated adsorption capacities (*q*_*e*_, cal) in the models were consistent with the experimental adsorption capacities (*q*_*e*_, exp). However, the pseudo-second-order model exhibited a higher correlation coefficient (*R*^2^) which was employed to estimate the consistency of the fitted models with experimental results than that of the pseudo-first-order model. Although, in consideration of a particular kinetic model, a relatively high *R*^2^ was not irrefragable evidence of a better fitting [49]. The Cr (VI) adsorption onto HTC-cornstalk in this work was considered that was apropos to the pseudo-second-order kinetic model.

### 3.6. Adsorption Isotherm Analysis

To investigate the Cr (VI) adsorbability of HTC, adsorption isotherms had been carried out with various initial concentrations of Cr (VI) (20, 30, 40, 50, 60, 70, 80, 90, and 100 mg/L) at different temperatures (35°C, 45°C, and 55°C).

**Figure 9:**
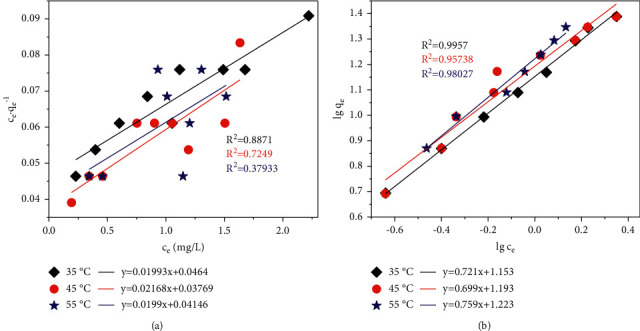
Langmuir isotherm model (a) and Freundlich isotherm model (b) for Cr (VI) adsorption onto HTC.

Results had been fitted with Langmuir (5) and Freundlich (6) isotherm models:

(5) $$\begin{array}{c} \frac{c_{e}}{q_{e}}=\frac{c_{e}}{q_{\max}}+\frac{1}{k_{1}q_{\max}}, \end{array}$$

(6) $$\begin{array}{c} \mathrm{lg}q_{e}=\mathrm{lg}\frac{c_{e}}{n}+\mathrm{lg}k, \end{array}$$

where *q*_*e*_ refers to the quantity of adsorbed metal ions per unit mass of adsorbent (mg/g), *c*_*e*_ represents the solute concentration in the bulk solution (mg/L) at equilibrium state, *q*_max_ (mg/g) and *k*_1_ represent the Langmuir constants related to the saturated metal ions adsorbability and the adsorption free energy, respectively, and the constants *k* and *n* in the Freundlich model represent the strength and the distribution of the adsorptive bonds. Experimental data fitted with Langmuir and Freundlich equations are shown in Figure 9. Furthermore, the fitted results are given in Table 4 in detail.

As revealed above, *R*^2^ values of higher than 0.9 were obtained in the Freundlich model which indicated an excellent consistency while fitting instead of many poor ones in the Langmuir model which were fitted at three different temperatures. Therefore, the Freundlich model was selected to express the Cr (VI) adsorption of HTC-cornstalk. Freundlich as an empirical equation had been employed to illustrate the exponential distribution sites, energies, and the heterogeneity of the adsorbent surface [50]. The adsorption capacities (K) increased with increasing temperatures. The values of *n* at different temperatures are greater than unity, indicating favorable adsorption of Cr (VI) [30, 51].

## 4. Conclusion

A high-efficiency HTC adsorbent was successfully synthesized by the one-step hydrothermal method for the uptake of Cr (VI). The adsorption property of HTC was studied by varying pH, HTC dosage, Cr (VI) concentration, and contact time. The Cr (VI) adsorption capability of HTC was pH-dependent, which was favored with a low pH range. The Cr (VI) removal efficiency of HTC reached 98% under optimal experiment conditions. A pseudo-second-order kinetic model could describe Cr (VI) adsorption behavior on HTC well, while the Freundlich adsorption isotherm was more suitable for Cr (VI) adsorption onto the adsorbent. Thus, the HTC was a potentially effective and sustainable adsorbent for application in Cr (VI) removal from wastewater

## Figures and Tables

**Table 1:** Textural characteristics.

Sample	Specific surface area (m^2^·g^−1^)	Total pore volume (cm^3^·g^−1^)	Micropore volume (cm^3^·g^−1^)	Mesopore rate (%)	Average pore size (nm)
HTC	85	0.42	0.003	99.93	3.41

**Table 2:** Ultimate analysis of samples.

Sample	Ultimate analysis (wt%)			
	C%	H%	O%^*∗*^	N%
Cornstalk	43.17	5.60	50.55	0.65
HTC-cornstalk	63.85	4.13	29.81	0.32

^*∗*^By difference.

**Table 3:** Adsorption kinetics parameters of Cr (VI) on HTC-cornstalk.

*q*_*e*_._exp_ (mg/g)	Pseudo-first-order kinetic model			Pseudo-second-order kinetic model		
	*q*_*e*_._cal_ (mg/g)	*k*_1_ (min^−1^)	*R* ^2^	*q*_*e*_._cal_ (mg/g)	*k*_2_ (g/mg min)	*R* ^2^
12.29	14.07	0.06	0.96	14.11	0.004	0.99

**Table 4:** The parameters of the isotherm adsorption model.

Temperature (°C)	Langmuir modes			Freundlich models		
	*q*_max_ (mg/g)	*K*_l_ (L/mg)	*R* ^2^	*K* (mg/g)	1/*n*	*R* ^2^
35	50.14640	0.42987	0.87281	14.49226	0.72818	0.98489
45	46.11816	0.57533	0.68560	15.63765	0.69809	0.95072
55	50.23585	0.48011	0.27588	17.50070	0.81531	0.91757

## Data Availability

The data used to support the findings of this study are included within the article.
